# Correlation of social smile symmetry with facial symmetry

**DOI:** 10.1186/s12903-023-03260-z

**Published:** 2023-08-14

**Authors:** Hoshyar Abbasi, Amin Golshah, Soraya Seifodini

**Affiliations:** 1 Department of Oral and Maxillofacial Surgery, School of Dentistry, Kermanshah University of Medical Sciences, Shariati Street, Kermanshah, 67139546581 Iran; 2 Department of Orthodontic, School of Dentistry, Kermanshah University of Medical Sciences, Shariati Street, Kermanshah, 67139546581 Iran; 3 School of Dentistry, Kermanshah University of Medical Sciences, Shariati Street, Kermanshah, 67139546581 Iran

**Keywords:** Smiling, Facial Asymmetry, Orthodontics

## Abstract

**Background:**

This study aimed to assess the correlation of social smile symmetry with facial symmetry.

**Methods:**

In this cross-sectional study, frontal view photographs were obtained from 169 eligible patients at rest and smiling with a camera at the level of their nose tip. Several landmarks were selected for facial symmetry and measured at rest and social smiling at the two sides of the face. The respective formula was used to calculate the asymmetry index (AI). The mean values for each AI were calculated, and the correlation between the criteria for a symmetric smile in a social smile with the criteria for facial symmetry, and the correlation between the difference in symmetry criteria at rest and social smiling with facial symmetry criteria were analyzed.

**Results:**

Significant correlations were noted between Oc-b AI (smile) and Sn-B (rest) facial AI (*P* = 0.046), An-a (smile) AI and Gn-a (rest) facial AI (*P* = 0.002), An-b (smile) AI and Sn-b (rest) facial AI (*P* < 0.001), Pog-a (smile) and Sn-a (rest) facial AI (*P* < 0.001), Nt-a (smile) and Sn-a (rest) facial AI (*P* < 0.001), Nt-b (smile) and Sn-b (rest) facial AI (*P* < 0.001), Ph-a (smile) and Sn-a (rest) facial AI (*P* < 0.001), Ph-b (smile) and Sn-b (smile) facial AI (*P* = 0.007), Oc-b AI (difference) and Gn-b (rest) facial AI (*P* = 0.031), Oc-Pog (difference) AI and Gn-b (rest) facial AI (*P* = 0.041), An-b (difference) AI and Sn-b (rest) facial AI (*P* < 0.001), Nt-a (difference) and Sn-a (rest) facial AI (*P* = 0.006), Nt-b (difference) and Sn-b (rest) facial AI (*P* < 0.001), and Ph-b (difference) and Sn-b (rest) facial AI (*P* < 0.001).

**Conclusions:**

A significant correlation exists between social smile symmetry and facial symmetry.

## Introduction

Esthetics is a philosophical, complex, and abstract concept. It can be a characteristic feature of humans, animals, locations, objects, or ideas, and creates a pleasant sense of satisfaction in the observers [1]. Improvement of smile esthetics is one of the most important motives for patients undergoing orthodontic treatment. Accordingly, creation of a beautiful smile along with a stable occlusion and efficient masticatory system is among the main goals of contemporary dentistry [2]. Smile design is currently an inseparable part of dental treatment planning. A successful outcome requires a correct understanding of the interactions of perioral facial structures such as the facial muscles, bones, temporomandibular joints, and also the gingiva and occlusion. A beautiful harmonious smile involves both facial and dental components [3]. Facial components include the facial hard and soft tissues, while dental components include the teeth and gingiva. Smile design should include assessment of both facial and dental components.

Facial esthetics is determined based on the standard principles of esthetics which include optimal arrangement of facial components, their symmetry, and facial proportions. Treatment planning for enhancement of facial esthetics is a multi-disciplinary approach, involving orthodontics, orthognathic surgery, periodontal surgery, cosmetic dentistry, and plastic surgery [4]. Some patients have an asymmetrical smile due to asymmetrical tension of the smile muscles. Also, in some cases, facial esthetic indices particularly midline may be different in smiling and at rest. Thus, it is important to assess whether facial asymmetry is correlated with smile asymmetry or not.

There are two features in the face that play a major role in smile design, namely the interpupillary line and the lips. As mentioned earlier, achieving a beautiful smile is one of the most important reasons for patients seeking dental treatment [5]. Knowledge about the features and details of the face in smiling position can help enhance facial esthetics. Smiles can be divided into two groups of social and enjoyment smiles [6]. A social smile is a voluntary and static facial expression, which involves the contraction of the levator muscles of the lip; the teeth and sometimes the gingiva are visible in a social smile [7]. According to Kiefer et al., [8] smile symmetry is a mini-esthetic component of dentofacial analysis. A symmetrical smile is more attractive. Also, an asymmetrical smile can suggest the presence of skeletal asymmetry. People with a visibly asymmetrical face often have a low quality of life. Thus, facial plastic surgery and orthognathic surgical procedures aim to minimize and clinically correct facial asymmetries as much as possible [8]. In some cases, the midline at rest does not coincide with the midline in social smile due to lack of symmetry [9]. Many patients demand a beautiful or at least a normal smile. To achieve this goal, dental clinicians should be able to recognize and correct unesthetic features of the face and smile.

Many researchers have addressed smile features in different populations, and have reported some criteria for a beautiful smile in the respective communities [10]. Coincidence of facial and dental midline is among such criteria [11]. Different methods are used for smile analysis [12]. The method proposed by Nakamura et al., [13] in 2001 is one such method. They used a frontal-view image of the face captured by a camera at the level of the nose tip (adjusted on a tripod). The focal spot was 100 mm, and the distance between the camera and object was 2 m. The person had to be in natural head position (NHP) during photography. The facial components were then analyzed on the photographs using a 2D analysis software.

Smile symmetry is an important prerequisite for a beautiful smile. Coincidence of dental midline and facial midline is imperative for a harmonious smile. Also, smile symmetry should be evaluated prior to orthodontic treatment and orthognathic surgery to predict the outcome of treatment and inform the patient about it. Although a minimal difference between the facial and dental midline is acceptable, a great difference adversely affects dentofacial esthetics. Also, the location and severity of asymmetry should be precisely determined prior to corrective surgery [14]. However, detection of asymmetry and offering effective treatment plans for its correction are challenging for dentists, orthodontists, and maxillofacial surgeons [15].

Dental literature is scarce regarding the relationship of social smile asymmetry and facial asymmetry. Thus, this study aimed to assess the correlation of social smile symmetry with facial symmetry. The null hypothesis was that facial symmetry would have no significant effect on smile symmetry.

## Methods

This cross-sectional study was conducted on 169 patients that were selected among those presenting to a dental clinic in Kermanshah city, Iran by convenience sampling. The study was designed according to the STROBE guidelines.

The sample size was calculated to be 169 according to a study by Jiménez-Castellanos et al., [16] assuming alpha = 0.05, d = 0.15, and the prevalence of midline deviation to be 0.361 according to the exact (Clopper-Pearson) method using PASS software.

The inclusion criteria were age over 18 years, no facial defect, no history of trauma, no neuromuscular disorder, no history of surgery or orthodontic treatment, class I molar relationship, absence of lip piercing, absence of visible facial deformity, no facial scar, and no congenital anomalies such as cleft lip and/or palate [13]. All patients signed informed consent forms prior to participation in the study.

### Photography

Two-dimensional photographs were obtained from the patients at rest and social smiling. To take the photographs at rest, the patients were asked to relax their lips, and look at their pupils in a mirror in front of them such that the head was in NHP. Also, the patients had no accessories on their face. For the social smile, the patients were asked to have a voluntary static smile. The levator muscles of the lip were contracted, and the teeth and sometimes the gingiva were visible.

The reference photographs were obtained by a Canon EOS 5DS R camera with 51 megapixels resolution, 8688 × 5792 pixels, and 72 dpi at 200 cm distance from the face with Canon Speedlite 600EX II external flash. To standardize the scales for the next steps, a 10-cm ruler was placed next to the face when taking the reference smile photographs.

### Assessment of facial and smile symmetry

Facial symmetry was assessed according to Nakamura et al. [13]. To analyze the face, a frontal-view image of the face was obtained by a camera placed on a tripod such that it was at the level of the nose tip. The focal spot was 100 mm, and the distance between the camera and object was 2 m. The patient was in NHP. The face was analyzed on photographs using a 2D analysis software.

### Facial landmarks

**Fig. 1:**
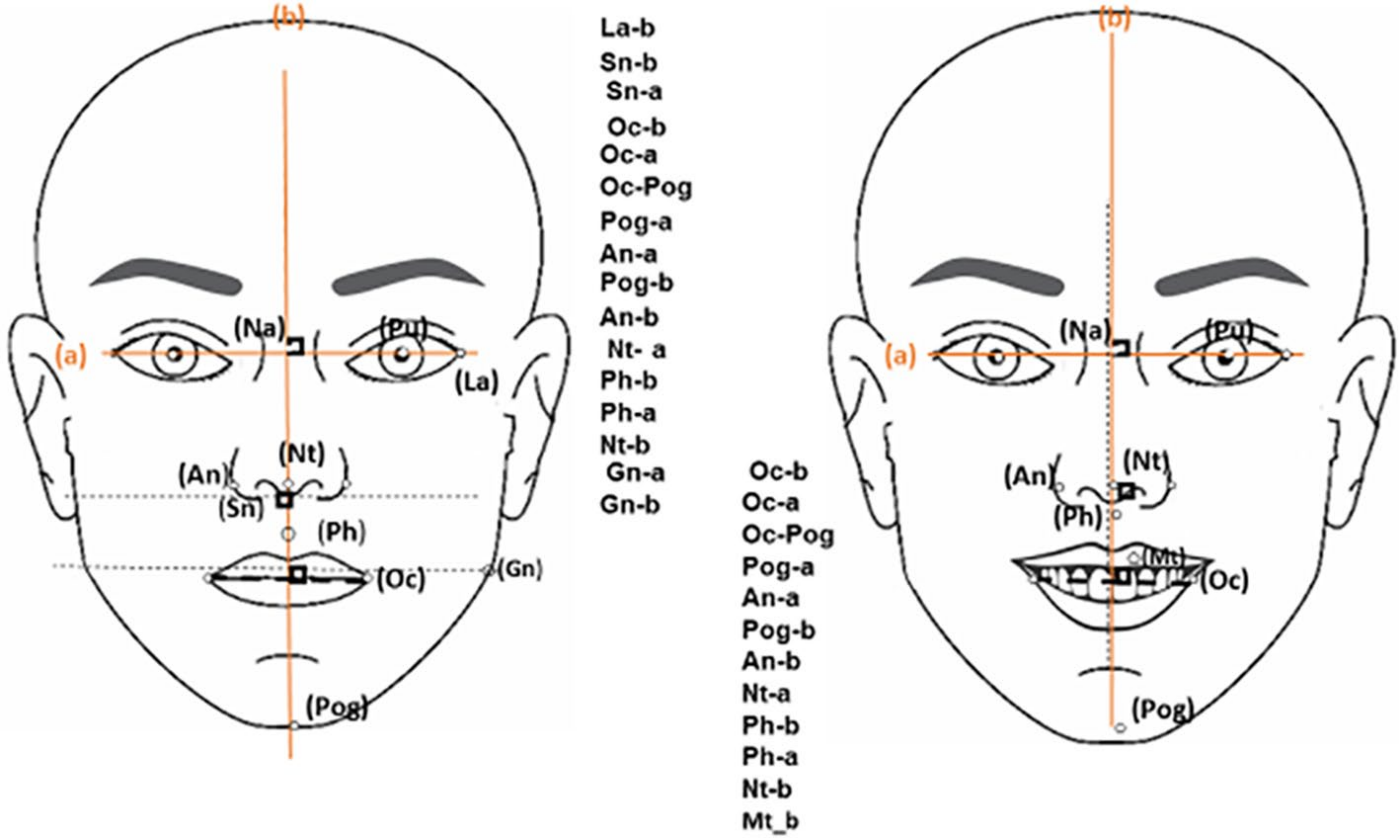
Facial symmetry criteria

Several landmarks were used to define facial and smile symmetry including the pupil (Pu), lateral angle of the eye (La), soft tissue nasion (Na), subnasale (Sn), oral commissure (Oc), tip of the nose (Nt), philtrum of the lip (Ph), ala of the nose (An), soft tissue pogonion (Pog), midline teeth (Mt), and Gonion (GN). The reference lines included the line connecting the right and left pupils as the horizontal line (a), and the line passing through Na and perpendicular to “a” as medial line of the face (b) or vertical line. The facial and smile criteria were measured at rest. The La-b, Sn-b, Sn-a, Oc-b, Oc-a, Oc-Pog, Pog-a, An-a, Pog-b, An-b, Nt-a, Ph-a, Ph-b, Nt-b, Gn-a, and Gn-b lines were measured bilaterally at rest (Fig. 1, Table 1).

**Table 1:** Definition of landmarks

Landmarks	Definition
A (Horizontal line)	A line connecting the right and left pupils
B (Vertical line)	A line passing from Na, which is perpendicular to “a” line
Pu (Pupil)	Eye pupil
Na (soft tissue nasion)	Deepest point of the nasal bridge
Sn (sub nasale	Midpoint of columella angle
Pog (soft tissue pogonion)	The most prominent mid-point of the chin
La (Lateral angel of the eye)	Lateral angle of the eye
Nt (Tip of the nose)	Most prominent point at the nasal tip
Oc (Oral commissure)	A point in labial commissure
An (Ala of nose)	The most lateral point in nasal alar contour
Gn (Gonion)	The most lateral point in the angle of mandible
Ph (Philtrum of lip)	Vertical groove at the mid-point of upper lip
Mt (Midline teeth)	A hypothetical line passing through the dental midline

### Assessment of facial symmetry

The facial symmetry criteria including Sn-b, Sn-a, La-b, Gn-b, and Gn-a were measured at rest, and the asymmetry index (AI) of each one was separately calculated.

The smile symmetry criteria including Pog-b, Pog-a, Oc-Pog, Oc-b, Oc-a, Nt-b, Nt-a, Ph-b, An-b, and An-a were measured bilaterally at rest and social smile. Also, the difference between the facial and dental midline was calculated (Mt-b).

The following formula was used to assess the AI for bilateral landmarks [13]:

$$\mathrm{AI}=|(\mathrm{R}-\mathrm{L})/(\mathrm{R}+\mathrm{L})| \times 100$$

A checklist was used to collect the required data. The first part of the checklist asked for the demographic information of patients such as age and sex. The second part included the symmetry criteria.

All measurements were made by a senior dental student. The variables related to 30 participants were assessed twice with a 2-week interval by the dental student, and the intraclass correlation coefficient was calculated to assess intra-examiner reliability. Also, the variables were measured once again by an orthodontist and compared with the values measured by the dental student. The inter-class correlation coefficient was then calculated.

### Statistical analysis

The mean and standard deviation values were calculated for each AI, and the correlation between asymmetry of the lines in social smile and at rest was calculated for each patient.

The correlation between the smile symmetry criteria in social smile and facial symmetry criteria, and also the correlation between the difference in smile symmetry criteria at rest and in social smile with facial symmetry criteria were analyzed. Also, the correlation between the smile asymmetry index criteria at rest and smiling was assessed.

Normal distribution of data was ensured by the Kolmogorov–Smirnov test (*P* > 0.05). Thus, the Pearson’s correlation test was applied to analyze the correlations between the variables. Statistical analyses were carried out using PASW version 18 (SPSS Inc., Released 2009. PASW Statistics for Windows, IL, USA) at 0.05 level of significance.

## Results

Of 169 participants, 141 (83.4%) were females and 28 (16.6%) were males. The mean age of participants was 30.21 ± 8.13 years. The mean age was 29.73 ± 8.30 years in females and 32.64 ± 6.82 years in males. The intraclass correlation coefficient for dental student was found to be > 0.950 indicating excellent intra-examiner reliability according to the Cicchetti’s classification. The inter-class correlation coefficient was calculated to be > 0.950, indicating excellent inter-examiner reliability.

Table 2 shows the significant correlations. Oc-a AI (difference), Oc-b AI (difference), Nt-b (difference), and Ph-b (difference) had no significant correlation with the facial symmetry criteria (*P* > 0.05). However, Oc-Pog AI (difference) (*P* = 0.038) and An-a AI (difference) (*P* = 0.030) had a significant correlation with Gn-b AI (rest). An-b AI (difference) (*P* < 0.001), Pog-a (difference) (*P* = 0.015) and Pog-b (difference) (*P* = 0.009) had a significant correlation with Sn-b (rest). Pog-a (difference) had a significant correlation with La-b AI (rest) (*P* = 0.033). Nt-a (difference) had a significant correlation with La-b AI (rest) (*P* = 0.031). Ph-a (difference) had a significant correlation with Sn-a (rest) (*P* < 0.001).

**Table 2:** Significant correlations between different indices and facial symmetry criteria

		Sn-a (rest)	Sn-b (rest)	La-b Asymmetry index (rest)	Gn-a Asymmetry index (rest)	Gn-b Asymmetry index (rest)
Oc-b Asymmetry index (difference)	Pearson Correlation	-.098	.102	.053	.011	**.166**
	*P-*value	.204	.185	.495	.888	**.031**
Oc-Pog Asymmetry index (difference)	Pearson Correlation	.012	-.043	-.005	.041	**.158**
	*P-*value	.881	.579	.944	.593	**.041**
An-b Asymmetry index (difference)	Pearson Correlation	-.094	**.343**	.129	.079	.023
	*P-*value	.223	** < 0.001**	.094	.307	.764
Nt-a (difference)	Pearson Correlation	**.211**	-.048	.021	.018	.102
	*P-*value	**.006**	.536	.783	.817	.186
Nt-b (difference)	Pearson Correlation	-.049	**.317**	**-.160**	.011	-.138
	*P-*value	.526	** < 0.001**	**.038**	.891	.074
Ph-a (difference)	Pearson Correlation	**.235**	-.085	.042	-.071	.051
	*P-*value	**.002**	.274	.586	.362	.511
Ph-b (difference)	Pearson Correlation	-.013	**.294**	-.143	**-.184**	.040
	*P-*value	.872	** < 0.001**	.063	**.016**	.601
Oc-b Asymmetry index (smile)	Pearson Correlation	.101	**.154**	-.019	-.038	.019
	*P-*value	.192	**.046**	.808	.621	.811
An-a Asymmetry index (smile)	Pearson Correlation	-.036	.034	.122	**.232**	.061
	*P-*value	.641	.662	.114	**.002**	.434
An-b Asymmetry index (smile)	Pearson Correlation	.048	**.302**	.071	-.021	.050
	*P-*value	.535	**.000**	.357	.782	.520
Pog-a (smile)	Pearson Correlation	**.591**	.084	-.001	.043	.027
	*P-*value	**.000**	.280	.985	.579	.727
Nt-a (smile)	Pearson Correlation	**.802**	.039	-.020	.050	-.025
	*P-*value	**.000**	.613	.793	.517	.748
Nt-b (smile)	Pearson Correlation	.035	**.479**	.145	.142	.124
	*P-*value	.649	**.000**	.060	.066	.109
Ph-a (smile)	Pearson Correlation	**.588**	.065	-.057	.030	.029
	*P-*value	**.000**	.402	.465	.703	.709
Ph-b (smile)	Pearson Correlation	-.007	**.207**	.050	.060	-.001
	*P-*value	.925	**.007**	.515	.438	.988

Table 3 shows insignificant correlations. As shown, the correlations between Oc-a AI (difference), An-a AI (difference), Pog-a (difference), Pog-b (difference), Mt-b (smile), Oc-a AI (smile), Oc-Pog AI (smile), and Pog-b (smile) with facial symmetry criteria were not statistically significant (*P* > 0.05).

**Table 3:** Insignificant correlations between different indices with facial symmetry criteria

		Sn-a (rest)	Sn-b (rest)	La-b Asymmetry index (rest)	Gn-a Asymmetry index (rest)	Gn-b Asymmetry index (rest)
Oc-a Asymmetry index (difference)	Pearson Correlation	-.043	.051	.022	.118	.006
	*P-*value	.580	.508	.781	.127	.937
An-a Asymmetry index (difference)	Pearson Correlation	-.011	.045	-.052	-.128	.011
	*P-*value	.887	.558	.504	.098	.891
Pog-a (difference)	Pearson Correlation	.114	-.138	-.056	-.145	.019
	*P-*value	.139	.073	.471	.059	.809
Pog-b (difference)	Pearson Correlation	-.073	.026	-.082	-.050	.125
	*P-*value	.349	.738	.291	.520	.105
Mt-b (smile)	Pearson Correlation	-.054	.109	-.088	.036	-.031
	*P-*value	.484	.160	.258	.641	.693
Oc-a Asymmetry index (smile)	Pearson Correlation	.016	-.020	.028	-.075	.052
	*P-*value	.841	.793	.716	.333	.505
Oc-Pog Asymmetry index (smile)	Pearson Correlation	.003	.052	-.016	.137	-.064
	*P-*value	.972	.505	.836	.077	.405
Pog-b (smile)	Pearson Correlation	-.044	.114	-.031	-.035	.014
	*P-*value	.572	.141	.686	.656	.855

Table 4 analyzes the association of Oc-a, Oc-b, Oc-Pog, An-a, An-b, Pog-a, Pog-b, Nt-a, Nt-b, Ph-a and Ph-b at rest and smiling. Significant correlations were noted between Oc-a asymmetry index (rest) and Oc-a asymmetry index (smile) (*P* = 0.021), Oc-b asymmetry index (rest) and Oc-b asymmetry index (smile) (*P* < 0.001), An-a asymmetry index (rest) and An-a asymmetry index (smile) (*P* = 0.047), An-b asymmetry index (rest) and An-b asymmetry index (smile) (*P* < 0.001), Pog-a (smile) and Pog-a (rest) (*P* < 0.001), Pog-b (smile) and Pog-b (rest) (*P* < 0.001), Nt-a (rest) and Nt-a (smile) (*P* < 0.001), Nt-b (rest) and Nt-b (smile) (*P* < 0.001), Ph-a (rest) and Ph-a (smile) (*P* < 0.001), and Ph-b (rest) and Ph-b (smile) (*P* < 0.001). The correlation between Oc-Pog asymmetry index (rest) and Oc-Pog asymmetry index (smile) was not significant (*P* = 0.071).

**Table 4:** Association of Oc-a, Oc-b, Oc-Pog, An-a, An-b, Pog-a, Pog-b, Nt-a, Nt-b, Ph-a and Ph-b at rest and smiling

Oc-a Asymmetry index (rest)	Oc-a Asymmetry index (smile)	
	Pearson Correlation	*P-*value
	.178	.021
Oc-b Asymmetry index (rest)	Oc-b Asymmetry index (smile)	
	Pearson Correlation	*P-*value
	.337	< 0.001
Oc-Pog Asymmetry index (rest)	Oc-Pog Asymmetry index (smile)	
	Pearson Correlation	*P-*value
	.139	.071
An-a Asymmetry index (rest)	An-a Asymmetry index (smile)	
	Pearson Correlation	*P-*value
	.153	.047
An-b Asymmetry index (rest)	An-b Asymmetry index (smile)	
	Pearson Correlation	*P-*value
	.398	< 0.001
Pog-a (rest)	Pog-a (smile)	
	Pearson Correlation	*P-*value
	.861	< 0.001
Pog-b (rest)	Pog-b (smile)	
	Pearson Correlation	*P-*value
	.501	< 0.001
Nt-a (rest)	Nt-a (Smile)	
	Pearson Correlation	*P-*value
	.822	< 0.001
Nt-b (rest)	Nt-b (smile)	
	Pearson Correlation	*P-*value
	.595	< 0.001
Ph-a (rest)	Ph-a (smile)	
	Pearson Correlation	*P-*value
	.771	< 0.001
Ph-b (rest)	Ph-b (smile)	
	Pearson Correlation	*P-*value
	.533	< 0.001

**Fig. 2:**
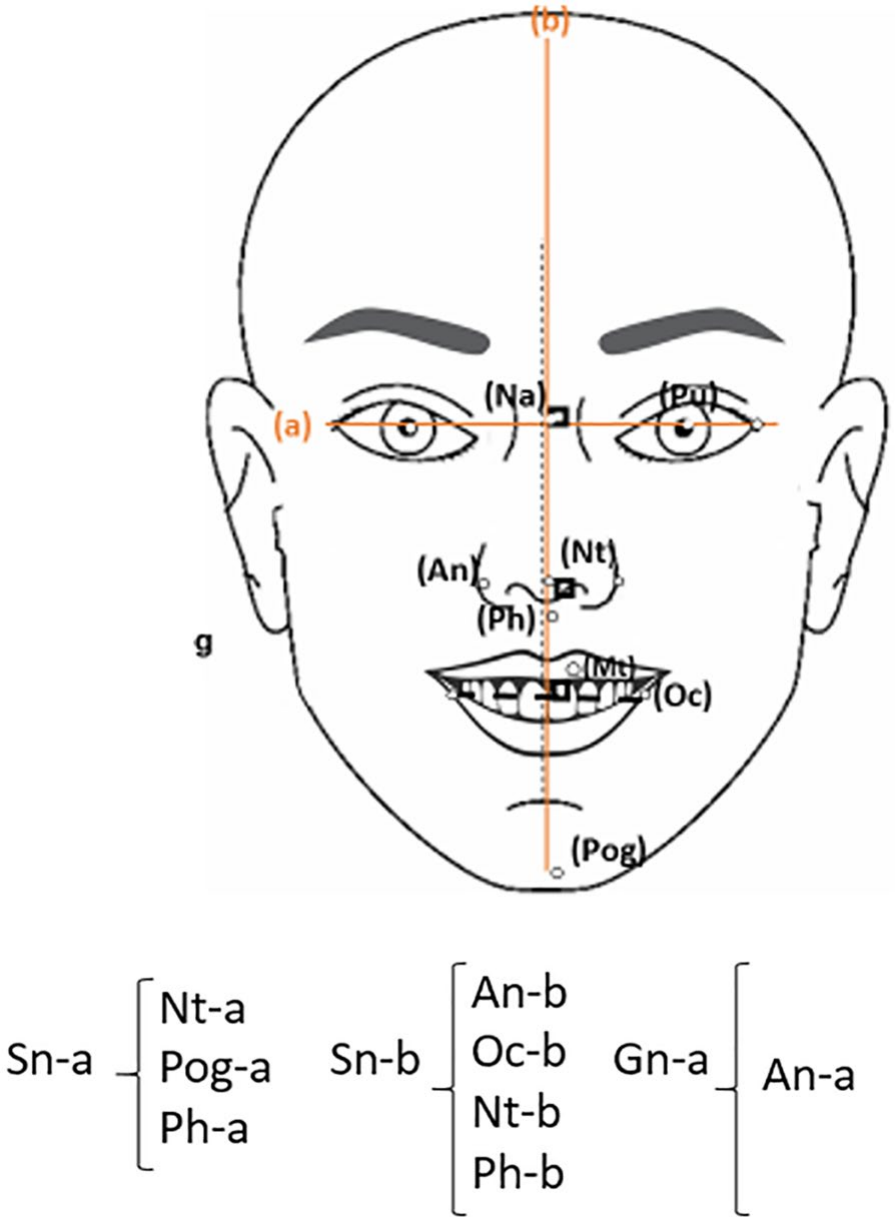
Correlation of smile symmetry criteria (AI for bilateral facial landmarks and the distance between the landmarks located at the midline and the midline) in smiling view with facial symmetry criteria

Figure 2 shows the correlation of smile symmetry criteria (AI for bilateral facial landmarks and the distance from the landmarks located at the midline) in smiling view with facial symmetry criteria.

**Fig. 3:**
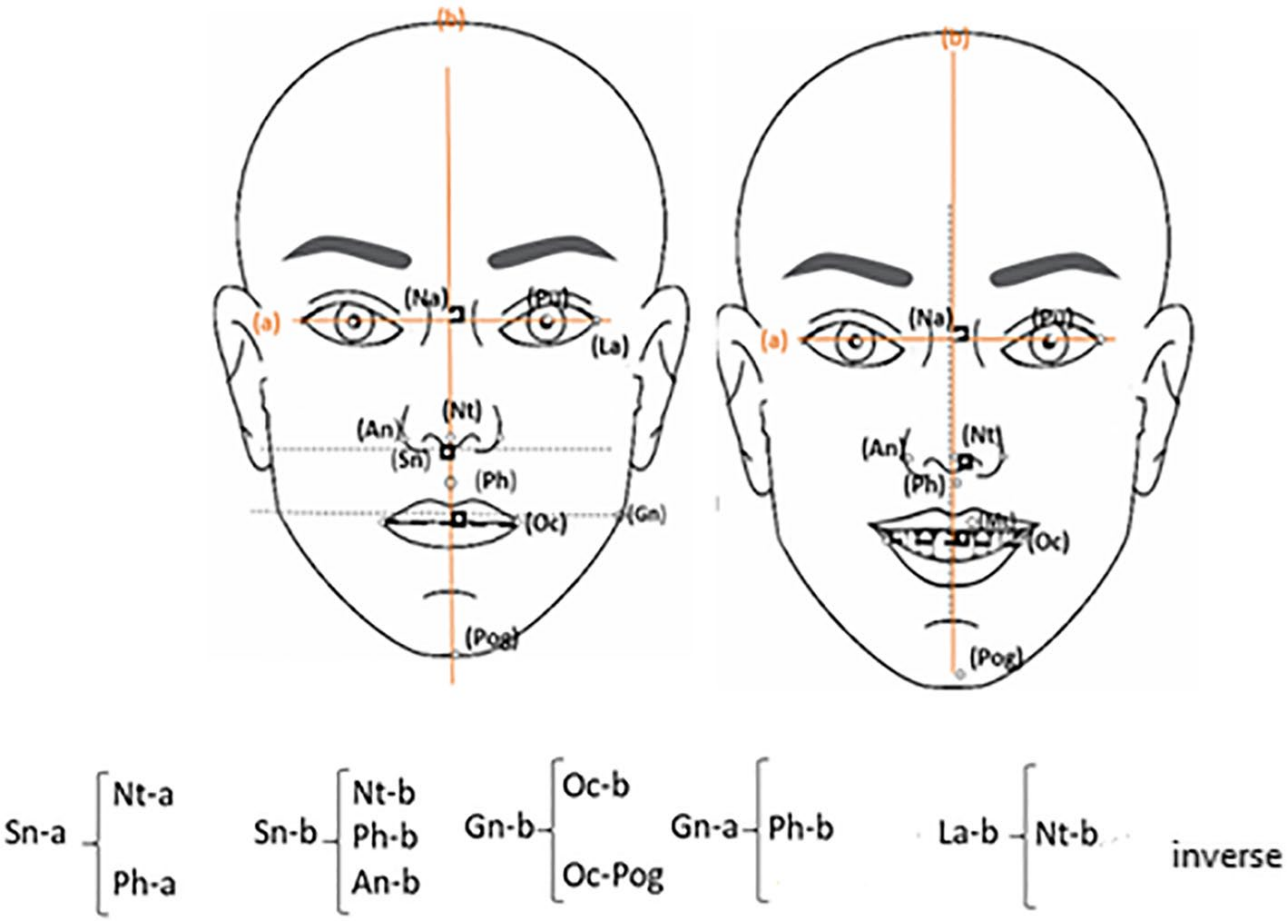
Correlation between the difference in smile symmetry criteria at rest and smiling views with facial symmetry criteria

Figure 3 shows the correlation between the difference in smile symmetry criteria at rest and smiling with facial symmetry criteria.

Table 5 compares the Gn-a AI (rest) with smile symmetry criteria. The mean Gn-a AI (rest) was significantly lower than Oc-b AI (smile) (*P* < 0.001) and An-b AI (smile) (*P* < 0.001).

**Table 5:** Comparison of the Gn-a AI (at rest) with smile symmetry indices

	Mean	Paired Differences		t	df	Sig. (2-tailed)^a^
		Mean	Std. Deviation			
Gn-a Asymmetry index (rest)	1.436	.198	1.615	1.597	168.000	.112
Oc-a Asymmetry index (smile)	1.238					
Gn-a Asymmetry index (rest)	1.436	-1.678	2.946	-7.403	168.000	.000
Oc-b Asymmetry index (smile)	3.114					
Gn-a Asymmetry index (rest)	1.436	.033	1.554	.274	168.000	.785
Oc-Pog Asymmetry index (smile)	1.404					
Gn-a Asymmetry index (rest)	1.436	.005	1.516	.044	168.000	.965
An-a Asymmetry index (smile)	1.431					
Gn-a Asymmetry index (rest)	1.436	-2.221	3.201	-9.021	168.000	.000
An-b Asymmetry index (smile)	3.657					

^a^Paired-Samples T-Test

Table 6 compares the mean Gn-b AI (rest) with smile symmetry criteria. The mean Gn-b AI (rest) was significantly greater than Oc-a AI (smile) (*P* < 0.001), Oc-Pog AI (smile) (*P* < 0.001), and An-a AI (smile) (*P* < 0.001), and significantly smaller than Oc-b AI (smile) (*P* = 0.030) and An-b AI (smile) (*P* < 0.001).

**Table 6:** Comparison of the mean Gn-b AI (rest) with smile symmetry indices

	Mean	Paired Differences		t	df	Sig. (2-tailed)^a^
		Mean	Std. Deviation			
Gn-b Asymmetry index (rest)	2.573	1.335	2.088	8.309	168	.000
Oc-a Asymmetry index (smile)	1.238					
Gn-b Asymmetry index (rest)	2.573	-0.541	3.217	-2.187	168	.030
Oc-b Asymmetry index (smile)	3.114					
Gn-b Asymmetry index (rest)	2.573	1.169	2.276	6.676	168	.000
Oc-Pog Asymmetry index (smile)	1.404					
Gn-b Asymmetry index (rest)	2.573	1.141	2.197	6.753	168	.000
An-a Asymmetry index (smile)	1.431					
Gn-b Asymmetry index (rest)	2.573	-1.085	3.412	-4.133	168	.000
An-b Asymmetry index (smile)	3.657					

^a^Paired-Samples T-Test

## Discussion

This study assessed the correlation of smile symmetry criteria and facial symmetry by two methods: First, the correlation between smile symmetry criteria in social smile with facial symmetry criteria was analyzed. Next, the correlation between the difference in smile symmetry criteria at rest and social smile with facial symmetry criteria was analyzed, which was a strength of this study. Also, correlations between smile symmetry criteria at rest and social smile were assessed.

Evidence shows that using different methods for assessment of facial symmetry results in different perceptions from the facial and smile symmetry [13]. Different techniques to determine the outline and facial angles yield different results. Thus, two different methods were adopted in the present study for this purpose [13, 15]. To the best of the authors’ knowledge, this study is the first to use facial asymmetry criteria for prediction of smile asymmetry. Since no previous study has addressed this topic, the present results cannot be compared with the results of other studies. Thus, we only discuss the possible reasons for the obtained results.

The present results indicated that facial symmetry criteria (La-b, Gn-a, Gn-b, Sb-b, and Sn-a) were symmetrical at rest. The results showed that sub-nasale asymmetry (a facial symmetry criterion) at rest had a direct correlation with oral commissure, tip of the nose, ala of the nose, and philtrum of the lip (smile symmetry criteria) when smiling. The length of middle third of the face at rest had a direct correlation with the distance from pogonion, tip of the nose, and philtrum of the lip to the horizontal line when smiling. The ramus height and angle of mandible symmetry at rest had a direct correlation with ala of the nose asymmetry when smiling. The asymmetry of angle of mandible at rest had a direct correlation with the asymmetry of oral commissure at rest and when smiling. Thus, the null hypothesis of the study was rejected.

Duran et al. [17] used 3D stereophotogrammetric images to assess social smile symmetry, and found asymmetries in social smile in different degrees and in different directions. They reported greater asymmetry in some specific areas particularly the mouth corners. They revealed the most common parameters responsible for social smile asymmetry. Smile asymmetry can be due to differences in muscle tonicity at the two sides of the face, yielding an asymmetric smile. Smile asymmetry in the vertical plane due to difference in lower lip tonicity at the two sides of the face may also lead to asymmetrical positioning of the mandibular right and left incisors.

Electromyography of facial muscles (circumoral) often gives a definite diagnosis regarding any muscular involvement [18]. Anatomical assessments have shown that after the formation of modiolus, the buccinator fibers extend to the upper and lower lips to form the peripheral part of the orbicularis oris muscle. The uppermost and lowermost fibers of the buccinator muscle are branched to enter the upper and lower lips. The risorius muscle fibers are also attached to the modiolus [19]. This fact explains the significant correlations of Oc and Gn with “b” line. Also, Gn showed a significant correlation with “b” line, and Oc showed a significant correlation with Pog, indicating harmonious contraction of mentalis and orbicularis oculi muscles in the modiolus [20]. The An and Sn nasal landmarks had a significant correlation with “b” line. Anatomically, the alar nasalis and depressor septi nasi muscle are connected to medial crus of the alar cartilage, and play a role in movements of the nasal alar [21]. Thus, the present results are justified. The reason for significant correlations of “b” and “a” lines with Nt-a and Sn-b may be the presence of origin of nasal levator muscles including levator labii and procerus that attach to the bone somewhere around the “a” line [22]. The significant correlation of nasal tip (Nt) and nasal alar from the “b” line may be related to the similar insertion site of compressor narium minor and alar nasalis muscles on the mesial crus [23].

Not having a suitable software program for automatic identification of landmarks and assessment of their symmetry at rest and when smiling was a limitation of this study. Also, we only evaluated 2D photographs of patients, and did not take 3D photographs, which was another limitation of this study. Moreover, all patients were Iranian, and recruited from a single center, and thus, may not be a representative sample of the entire population of Iran. To assess the correlation between soft tissue and hard tissue symmetry, the facial soft tissue scans should be compared with hard tissue scans (such as computed tomography), which was not performed in this study. Future studies are recommended to use 3D scans of patients and evaluate different ethnic and racial groups.

Statistical analysis showed that only Oc-Pog (distance between the lip commissure and most anterior point of the mid-chin) was not significantly different at rest and smiling. This finding may be due to the absence of a muscular correlation between the abovementioned two points. Orbicularis oris, depressor anguli, and risorius muscles are involved in lip commissure movements [24, 25]; whereas, the Pog point only depends on the movement of mentalis muscle [26]. Accordingly, it may be concluded that the distance between the lip commissure and pogonion cannot indicate asymmetry in smiling compared with resting position.

All variables of Oc-a (distance between the lip commissure and line a), Oc-b (distance between the lip commissure and line b), An-a (distance between the most lateral point in the nasal alar contour and line a), An-b (distance between the most lateral point in the nasal alar contour and line b), Pog-a (distance between the most anterior point of the mid-chin and line a), Pog-b (distance between the most anterior point of the mid-chin and line b), Nt-a (distance between the most prominent point of the nose tip and line a), Nt-b (distance between the most prominent point of the nose tip and line b), Ph-a (distance between the vertical groove in the middle of the upper lip and line a), and Ph-b (distance between the vertical groove in the middle of the upper lip and line b) were significantly different at rest and smiling. Presence of a significant difference in distance between the abovementioned landmarks and reference lines a and b indicates the significance of these landmarks in detection of smile asymmetry compared with resting position. The muscles involved in smile include the risorius, depressor anguli, levator anguli, orbicularis oris, zygomaticus minor and zygomaticus major. Although the mentalis muscle which moves the pogonion does not have a direct role in smile, Root et al. [27] demonstrated that it has a role in showing horror. The present results revealed that the distance between the pogonion and the two reference lines was significantly different in smiling and at rest. The present results also indicated that the distance between the pogonion and the two reference lines was significantly different in smiling and at rest. Thus, it may have a role in determining facial asymmetry in smiling and at rest. Evidence shows that the internal fascicles of depressor septi nasi muscle and nasal tip depression are correlated with changes in the alar base or nasal alar alterations when smiling [28]. These results were in line with the present findings. However, this muscle, which has a role in moving the nose tip and nasal alar, is not among the main smile muscles, and can be a debating topic in the present study.

## Conclusion

The present results revealed significant correlations between some landmarks of facial asymmetry with smile asymmetry including Sn-a with Nt-a, Ph-a, and pog-a, Sn-b with An-b, Oc-b, Nt-b, Pog-a and Ph-b, Gn-a with An-a, Gn-b with Oc-pog and An-a, and La-b with Nt-a and Pog-a. Correlations were also noted between the asymmetry index of some smile symmetry criteria in smiling and at rest (Oc-a, Oc-b, An-a, An-b, Pog-a, Pog-b, Nt-a, Nt-b, Ph-a, and Ph-b). Thus, it may be concluded that social smile symmetry has a correlation with facial symmetry.

## Data Availability

The data used to support the findings of this study were supplied by corresponding author under license and data will be available on request. Requests for access to these data should be made to corresponding author.

## Abbreviations

AI: Asymmetry index
NHP: Natural head position
